# Organic acid blend supplementation increases butyrate and acetate production in  *Salmonella enterica* serovar Typhimurium challenged broilers

**DOI:** 10.1371/journal.pone.0232831

**Published:** 2020-06-04

**Authors:** Mashael R. Aljumaah, Manal M. Alkhulaifi, Alaeldein M. Abudabos, Abdulaziz Alabdullatifb, Aarif H. El-Mubarak, Ali R. Al Suliman, Dragana Stanley

**Affiliations:** 1 Department of Botany and Microbiology, College of Science, King Saud University, Riyadh, Saudi Arabia; 2 Department of Animal Production, College of Food and Agriculture Sciences, King Saud University, Riyadh, Saudi Arabia; 3 Department of Plant Protection, College of Food and Agriculture Sciences, King Saud University, Riyadh, Saudi Arabia; 4 Department of Biochemistry and Molecular Biology, Faculty of Science, University of Gezira, Medani, Sudan; 5 King Abulaziz City for Science and Technology, Riyadh, Saudi Arabia; 6 Institute for Future Farming Systems, Central Queensland University, Rockhampton, Queensland, Australia; Tokat Gaziosmanpasa University, TURKEY

## Abstract

The burden of enteric pathogens in poultry is growing after the ban of antibiotic use in animal production. Organic acids gained attention as a possible alternative to antibiotics due to their antimicrobial activities, improved nutrient metabolism and performance. The current study was conducted to evaluate the effectiveness of organic acid blend on broilers cecal microbiota, histomorphometric measurements, and short-chain fatty acid production in *Salmonella enterica* serovar Typhimurium challenge model. Birds were divided into four treatments, including a negative control, positive control challenged with *S*. Typhimurium, group supplemented with an organic acid blend, and birds supplemented with organic acid blend and *Salmonella* challenged. Results illustrate significant differences in feed conversion ratios and production efficiency factor between treatment groups, however, the influence of organic acid supplement was marginal. Organic acid blend significantly increased cecal acetic and butyric acids concentrations when compared to unsupplemented groups and resulted in minor alterations of intestinal bacterial communities.

## Introduction

Antibiotic growth promoters (AGPs) have been used in the animal industry for several decades in various countries, to improve the production rate and reduce mortality [1]. However, European Union countries banned the use of AGPs in food animal production in 1995, due to the increasing concerns of high spread of antimicrobial resistance genes [2]. This action was followed by several other countries and created an urgent need for alternative solutions.

In recent years, organic acids gained attention as a possible alternative for AGPs, especially in the poultry industry. Organic acids demonstrated great potential as antimicrobial agents against a wide range of intestinal pathogens [3], especially after European Union approved the use of organic acids and their salts in poultry production as safe AGP alternatives [4]. Organic acids used in poultry were reported to exhibit antimicrobial actions against a wide range of intestinal pathogens, enhance nutrient metabolism, and improve performance [3,5]. The efficacy of an organic acid is influenced by several factors such as the chemical composition and form, pKa-value, with higher pKa values associated with better antibacterial activity, molecular weight and targeted microorganism control [6,7].

Adil et al. [4] tested the effect of multiple organic acids (butyric, fumaric and lactic acid) on poultry at varying concentrations, demonstrating beneficial influence on bird’s body weight gain, feed conversion ratio and increased villus height in the small intestine irrespective of type and concentration of acid used. Abudabos et al. [8] reported that the effect of organic acid blend in *S*. Typhimurium challenged broilers was similar to the effect of antibiotic avilamycin. Van Immerseel et al. [9] and Fernandez-Rubio et al. [10] showed that the supplementation with butyric acid-based feed additives reduced poultry susceptibility and shedding of *Salmonella* infection, thereby indirectly reducing the contamination of litter with pathogens.

Normal gut microbiota harbours millions of genes that support vital functions, including the functions the host is incapable of performing. As a consequence, the microbiota has a large influence on many important aspects of the development of the host [11]. Therefore, the extensive study of the chicken gut microbiota may give answers to a better understanding of how diet supplementations and environmental factors can be used to benefit the host [12,13].

Our present study was conducted to evaluate the effectiveness of organic acid blend using next-generation sequencing-based microbiota analysis, in combination with histomorphometric measurements and SCFA production profile in broiler chickens under *S*. Typhimurium challenge.

## Materials and methods

### Birds management and dietary treatments

The study was conducted on 100 one-day-old broiler chicks (Ross 308). Broilers chicks were randomly allocated into four dietary treatment groups; each treatment was further separated into five replicates with five birds per replica. The growth experiment was conducted for five weeks, with a standard starter period (1-21d) and finisher period diets (21-35d). Birds were raised under the strict biosecurity and hygienic settings in controlled light and temperature, *ad libitum* access to feed and water, and were kept on a 24 h light schedule.

A standard diet with isocaloric and isonitrogenous contents was offered in the mashed formula in both starter and finisher periods. Corn and soybean meal (corn-SBM) diet was formulated as per Ross 308 suggested recommendation, to meet or exceed recommendations in commercial settings in Saudi Arabia (Table 1). Organic acid (OA) blend was added into two groups over the top. Upon arrival, chicks were randomly distributed to one of the four treatment groups as follows: Treatment 1: negative control (no challenge, no OA); Treatment 2: positive control (*S*. Typhimurium challenge); Treatment 3: organic acid blend (OA -3.0 g/kg); Treatment 4: both OA (3.0 g/kg) and *S*. Typhimurium challenge. The commercial OA we used was Fysal^®^ Fit 4 (Trouw Nutrition, Ireland). Fysal® Fit 4 is a blend of short and medium-chain fatty acids and a low dose of ß1-4 mannobiose. The manufacturer does not provide detailed blend information, however, the product is widely used in poultry.

**Table 1:** Ingredients and calculated nutrient analysis of broilers starter and finisher diets.

Ingredients		
	Starter	Finisher
Yellow corn	50.635	65.39
Soybean meal	42.40	20.70
Wheat bran	0.00	0.60
Corn Gluten meal	0.00	0.70
Choline chloride CL 60	0.05	0.05
Corn oil	3.60	3.00
Dicalcuim Phosphate DCP	1.270	1.027
Ground Limestone	1.080	1.04
Salt	0.300	0.30
Phytase xp 10000 TPT	0.005	0.005
DL-methionine	0.295	0.22
Lysine-HCL	0.080	0.36
Threonine	0.085	0.11
Vitamin- Mineral premix^1^	0.200	0.200
Total	100	100
Analysis		
ME, kcal/kg	3000	3200
Crude protein, %	23.0	19.5
Non phytate P, %	0.48	0.359
Calcium, %	0.96	0.81
D. Lysine, %	1.28	1.03
Sulfur amino acids, %	0.85	0.8
Threonine, %	0.86	0.69

^1^Vitamin-mineral premix contains in the following per kg: vitamin A, 12000000 IU; vitamin D3, 5000000 IU; vitamin E, 80000 IU; vitamin K3, 3200 mg; vitamin B1, 3200 mg; vitamin B_2_, 8600 mg; vitamin B_3_, 65000 mg; pantothenic acid, 20000 mg; vitamin B_6_, 4300 mg; biotin 220 mg; antioxidant(BHA+BHT), 50000 mg; B_9_, 2200 mg; B_12_, 17 mg; copper, 16000 mg; iodine, 1250 mg; iron, 20000 mg; manganese, 120000 mg; selenium, 300 mg, and zinc, 110000 mg.

At the end of the first week (7d), birds from T2 and T4 were orally challenged with 1 ml 3 x10^9^ CFU *Salmonella enterica* subsp. *enterica*, serovar Typhimurium (*S*. Typhimurium) (ATCC# 14028). The challenge inoculum was prepared as described previously [14].

### Performance measurements

Overall performance was measured on a weekly basis, from week 1 to week 5 (35 days), feed intake (FI) was determined by subtracting the entire rejected feed from the total feed. Average body weight gain (BWG) and adjusted feed conversion ratios (FCR) were corrected for the bird’s mortality and computed for each group. Production efficiency factor (PEF) was calculated using the following formula:

$$PEF=\frac{Livability\times Live\;weight\;(kg)}{Age\;in\;days\times FCR}\times 100$$

### Analysis of broilers cecal microbiota composition

On day 40, cecum contents were collected randomly from 14 birds from each of the treatment groups. Samples were directly transported to the lab in sterile containers under ice and stored at -80°C. Total DNA was extracted using a protocol designed and described previously by Stanley et al. [15] with minor modifications. The quality and quantity of DNA were confirmed using a Nanodrop spectrophotometer. Hypervariable regions V3–V4 of the 16S rRNA gene were amplified and sequenced to determine the cecal microbiota using primers; forward ACTCCTACGGGAGGCAGCAG, reverse GGACTACHVGGGTWTCTAAT. Primers used were uniquely barcoded and contained Illumina spacers and sequencing linkers, using an approach that has been proposed by Fadrosh et al. [16]. The sequencing library was prepared by following the manufacturer's protocol (Illumina Inc., San Diego, CA, USA). Sequencing was performed using the Illumina MiSeq 2x300 bp paired-end sequencing.

### Bioinformatics analysis

Illumina MiSeq sequencing generated reads were primarily investigated using Quantitative Insights Into Microbial Ecology (QIIME v.1.9.1) [17]. Fastq-Join algorithm was used to join pair-end sequences allowing no mismatches within the overlap region. Only sequences with greater than 20 Phred quality threshold were taken in further analysis. Using UCLUST algorithm, sequences were clustered and grouped into operational taxonomic units OTUs at 97% match [18], and inspected for chimeric sequences using Pintail [19]. All taxonomic assignments were performed in QIIME against the GreenGenes database using QIIME default arguments [20]. Calculation of Weighted and Unweighted Unifrac distance matrixes was done using QIIME, with 99,999. OTUs with a relative abundance of less than 0.01% were removed. After quality filtering, there were 50 cecum samples that were sequenced to sufficient depth and sequence quality. Data analysis was completed using Hellinger transformed rarefied data [21]. Data visualization were done via Calypso (http://cgenome.net/calypso/) [22]. Chao1, Shannon, Richness, Evenness and Simpson indexes were used to measure Alpha diversity. Figure comparing relative abundance shows the untransformed version of the data. ANOVA was used to detect the significance of univariates. Multivariate data visualisation and multivariate statistical testing were inspected by applying the supervised multivariate redundancy analysis (RDA) using 999 permutations. Complete sequencing dataset is accessible on the MG-RAST public server (http://metagenomics.anl.gov/) with library ID mgl758080.

### Extraction and analysis of short-chain fatty acids

On day 40, cecal contents were collected from 56 birds (14 birds per treatment) for SCFAs analysis. An acidified water-extraction protocol described by Zhao et al. [23] was used for SCFAs extraction with minor alterations. Acetic acid (99.9%), propionic acid (99.9%), butyric acid (99.5%), *i*-butyric acid (99.0%), n-valeric acid (99.3%) and *i-*valeric acid (99.0%) were obtained from Dr Ehrenstorfer (Augsburg, Germany), and adjusted to 1000 ug/ml (ppm) in methanol. Gas chromatograph/Mass spectrometer (GC/MS) on an Agilent (Palo Alto, CA) 7890A with an Agilent 350°C separation column (30 m x 250 μm x 0.25 μm) was used. The retention time of each SCFA was defined using total ion chromatogram (TIC) scan mode. SCFAs separation was done following methods described previously [24–27]. A single ion monitoring (SIM) mode was used to operate the GC/MS following instrumental parameters. Oven programme was 18.133 min starting on 50°C for 1 min, then 6°C per min to 100°C for 1 min, next 25°C per min to 270°C for 1 min, and 2 min post run 300°C. Sample of 2 μL was injected at 250°C using split-less mode and helium carrier gas. The pressure was fixed on 11.747 psi at 24.4 mL/min total flow. Calibration curves were constructed from a standard stock concentration of 1000 ppm of all six tested acids.

### Histological analysis

Ileum tissue was collected aseptically at day 40, from six birds per treatment (two centimetres from the mid-section) and fixed immediately in 10% buffered formaldehyde, followed by dehydration and paraffin embedding. Sections were cut in 5 μm and were stained by haematoxylin and eosin (H&E) stain. Images were scanned with a Nikon Eclipse Ni-U microscope with the camera (Nikon, Tokyo, Japan) at magnifications power of (4x, 10x, 40x). Histomorphometric measurements, villi height, width, and total surface area were assessed based on a minimum of 10 well-oriented villi/section using an IX71 Inverted Olympus Microscope with a PC-based image analysis system (Olympus DP72 microscope digital camera; Olympus NV, Aartselaar, Belgium), and analysis was done using CellSens digital imaging software. Villus surface area was calculated using the formula:

VillusSurfaceArea:[2π×(villuswidth/2)×villuslength]

### Statistical analysis

Performance and sampling data were analysed using ANOVA for a complete randomized block design, using SAS software (SAS, 2009) general linear models (GLM) procedure. Least significant difference (LSD) test was used to compare the group means when the treatment impact was significant at *P*-value <0.05.

### Ethical statement

This trial was performed under project number (KSU-SE-18-38) with the approval of the Research Ethics Committee, Deanship of Scientific Research, Vice-Rectorate for Graduate Studies & Scientific Research at King Saud University. All methods were performed in accordance with the Gloucestershire County Council’s (GCC) Animal Welfare Act endorsed by Saudi Arabia and approved in Royal Decree No. (M / 44).

## Results

### Organic acid supplementation effect on growth performance

Cumulative performance measurements were calculated at the end of the finisher period (21-35d) including average feed intake (FI), body weight gain (BWG), feed conversion ratio (FCR), and production efficiency factor (PEF) (Table 2). A significant impact was observed on average FCR and PEF (*P* = 0.012, *P* = 0.047 respectively), were birds under *S*. Typhimurium challenge were characterised with the highest FCR (1.646). Broilers that were under *S*. Typhimurium challenge and had their diet supplemented with OA showed marginally lower FCR compared to the unsupplemented *S*. Typhimurium challenged group. The highest PEF values were detected in T1 followed by T4 and T3, while the lowest value was recorded in T2 (*S*. Typhimurium). There were no mortalities in T1, 10.4% in T2, 0.4% in T3 and 6% in T4. The mortality has taken into account how long the bird lived (the age at the time of death).

**Table 2:** Cumulative Feed Intake (FI), body weight gain (BWG), feed conversion ratio (FCR), and production efficiency factor (PEF) of broilers given different diet supplementation during the finisher period (21-35d).

Treatment group	Performance			
	FI, g	BWG, g	FCR, g:g	PEF
T1	1597.4	1128.6^a^	1.413^c^	390.4^a^
T2	1661.5	1014.3^b^	1.646^a^	318.4^b^
T3	1589.9	1073.1^a^^b^	1.484^b^^c^	356.6^a^^b^
T4	1669.9	1075.5^a^^b^	1.554^a^^b^	348.9^a^^b^
**SEM±**	**±39.77**	**±31.73**	±0.038	±14.07
***p-value***	0.41^NS^	0.0211*	0.012**	0.047*

T1: Negative Control, basal diet, unchallenged; T2: Positive Control, *S*. Typhimurium Challenge; T3: OA, unchallenged and T4: OA, *S*. Typhimurium Challenge.

BWG, body weight gain; FI, feed intake; FCR, feed conversion ratio; PEF, production efficiency factor.

^abcd^Means in the column with different superscripts differ significantly.

NS, not significant.

### Organic acid effect on ileum histomorphological measurements

The impact of organic acid supplementation on ileum histomorphometric measurements including villus length (L), width (W), and surface area (SA) in broilers at (40 d) are presented in Table 3. *S*. Typhimurium challenge was associated with a significant decrease in villus length and intestinal surface area. Birds from T4 (challenged and supplemented) had significantly higher villus length than challenged group T2, while there were no differences in width and surface area.

**Table 3:** Effect of different dietary supplementation and bacterial challenges on ileum histomorphometric measurements of broilers at (40 d).

Treatment group	Histomorphometric Measurements		
	L (μm)	W (μm)	SA (μm)
T1	624.8^a^	75.9	0.147^a^
T2	535.2^b^	63.3	0.109^b^
T3	603.9^a^^b^	75.7	0.152^a^
T4	622.7^a^	70.3	0.139^a^^b^
**SEM**^1^	**±18.83**	**±**4.08	±0.0100
***p-Value***	0.0024***	0.1002	0.012**

T1: Negative Control, basal diet, unchallenged; T2: Positive Control, *S*. Typhimurium Challenge; T3: OA, unchallenged and T4: OA, *S*. Typhimurium Challenge.

L: villus length; W: villi width; SA: surface area

^1^ SEM: standard error of the mean

^abc^ Means values within a column with different superscripts are significantly different

*, *P* <0.05

**, *P* <0.01

***, *P* <0.001, NS, not significant.

### Microbiota structure overview

**Fig 1:**
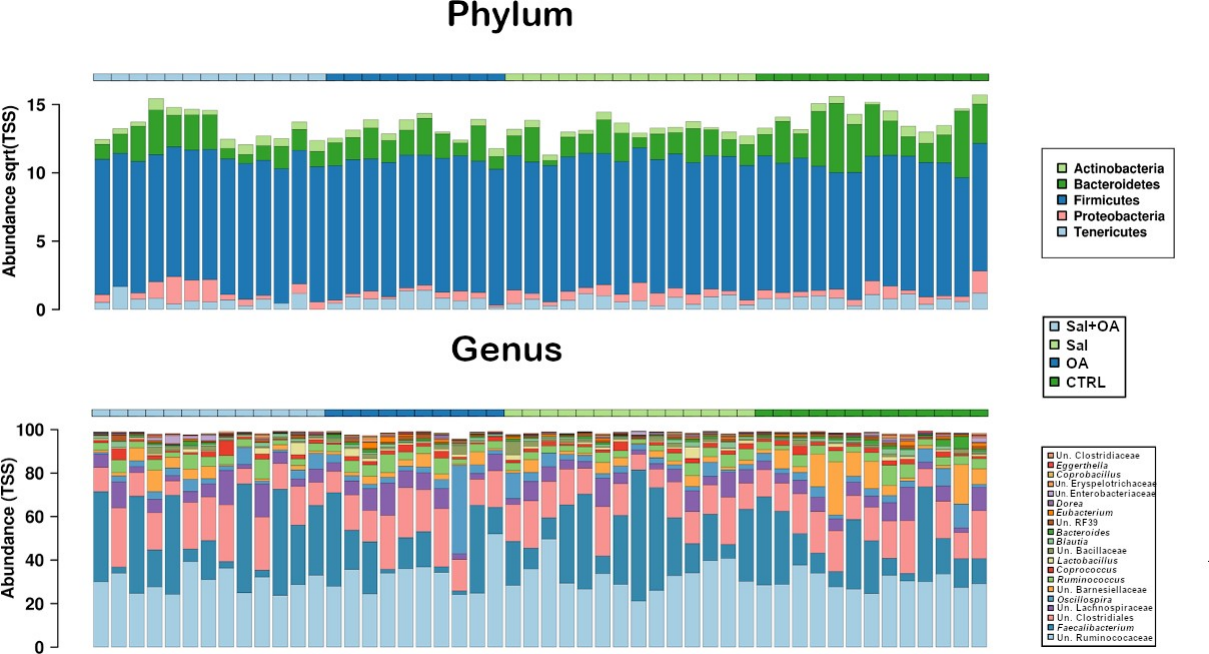
Overall microbiota composition variation of broilers within different groups (top) the most dominant phyla, and (bottom) the 20 most abundant genera.

The sequencing of 16S rRNA gene revealed the dominance of five main phyla, where Firmicutes were highly dominant, followed by abundant Bacteroidetes, along with Actinobacteria, Tenericutes, and Proteobacteria (Fig 1A). The most abundant culturable genera identified from cecal microbiota reads were *Faecalibacterium* and *Oscillospira* followed by *Ruminococcus*, *Coprococcus*, *Lactobacillus*, *Blautla*, *Bacteroides*, *Eubacterium*, *Dorea*, *Coprobacillus*, and *Eggerthella*. The remaining identified bacteria belonged to unclassified genera as presented in Fig 1B.

### Cecal microbiota composition

**Fig 2:**
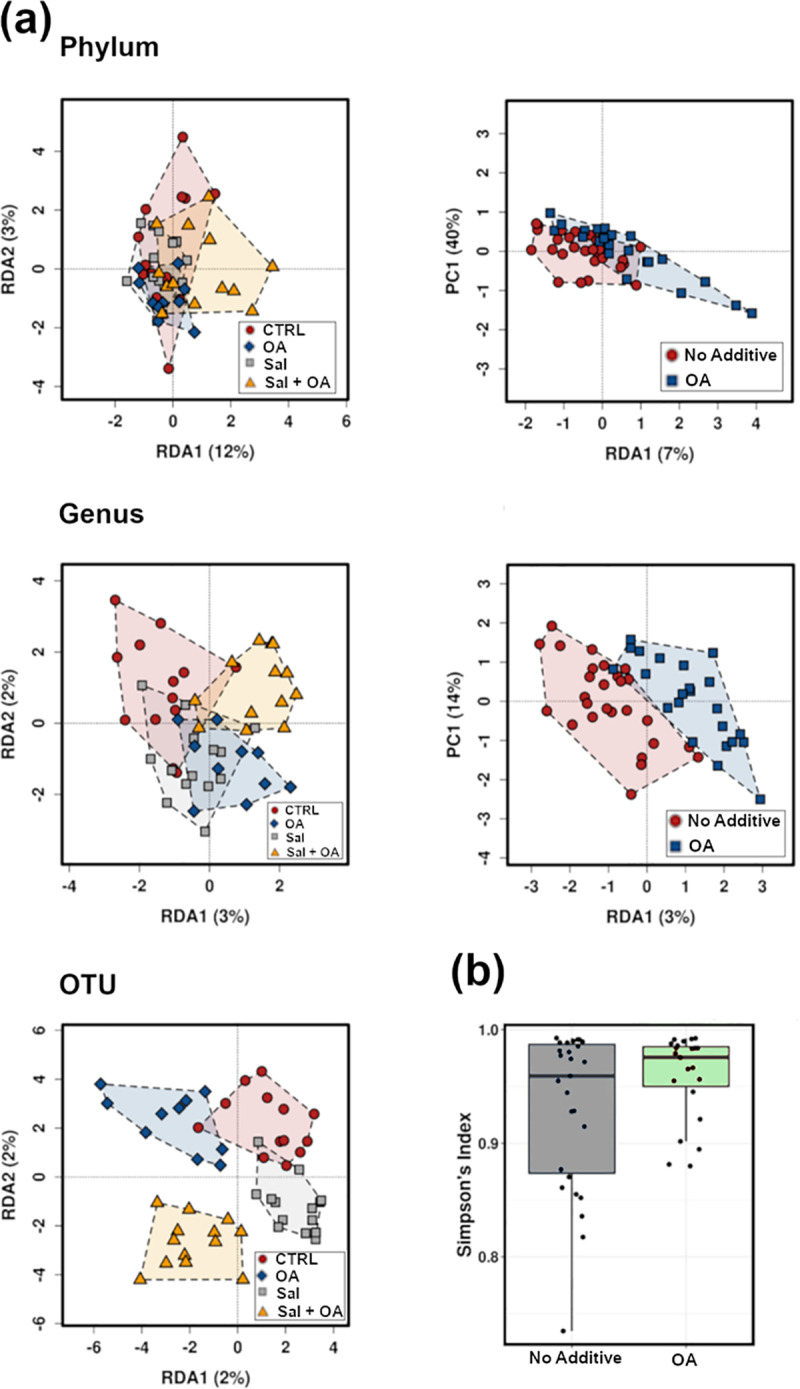
Multivariate redundancy analysis (RDA) plots at a phylum, genus, and OTU levels. Showing OA supplementation significantly impacting beta diversity at phylum and genus level (a), Alpha diversity marginally affected by organic acid OA (*P* = 0.0.81) (b), CTR: negative control.

Microbiota diversity analysis revealed no significant differences between bird treatment groups (T1: negative control, T2: positive control, *S*. Typhimurium challenged, T3: OA (3.0 g/kg), T4: OA (3.0 g/kg) and *S*. Typhimurium challenged) using several alpha diversity parameters including Shannon, Richness, Chao1, Evenness, and Simpson indexes (Fig 1A). Likewise, when we separated birds based on the two main factors of the trial (*S*. Typhimurium challenge and OA supplementation) we detected a marginal increase in the number of species present and their Simpson's diversity index marginally influenced by organic acid supplementation (*P* = 0.081) (Fig 2B).

Multivariate RDA analysis showed highly significant differences in microbiota composition at a phylum level (*P* = 0.003) between the 4 groups with no significant difference by *Salmonella* (*P* = 0.263) but with a significant effect of OA blend as a factor (*P* = 0.014). This was confirmed using 2-way PERMANOVA (Primer-e) with 99999 permutations, showing the significant influence of OA supplement (*P* = 0.012), the marginal influence of *Salmonella* (*P* = 0.059) and significant interactions (*P* = 0.027) between *Salmonella* challenge and OA.

At the genus level, RDA showed insignificant differences between the 4 groups (*P* = 0.159) and *Salmonella* (*P* = 0.513) and significant influence of OA blend (*P* = 0.030). Genus level significance of organic acid blend was not confirmed with 2-way PERMANOVA (*P* = 0.162). At an OTU level, both RDA analysis and 2-way PERMANOVA showed no significant difference between either all groups, individual factors or interactions.

**Fig 3:**
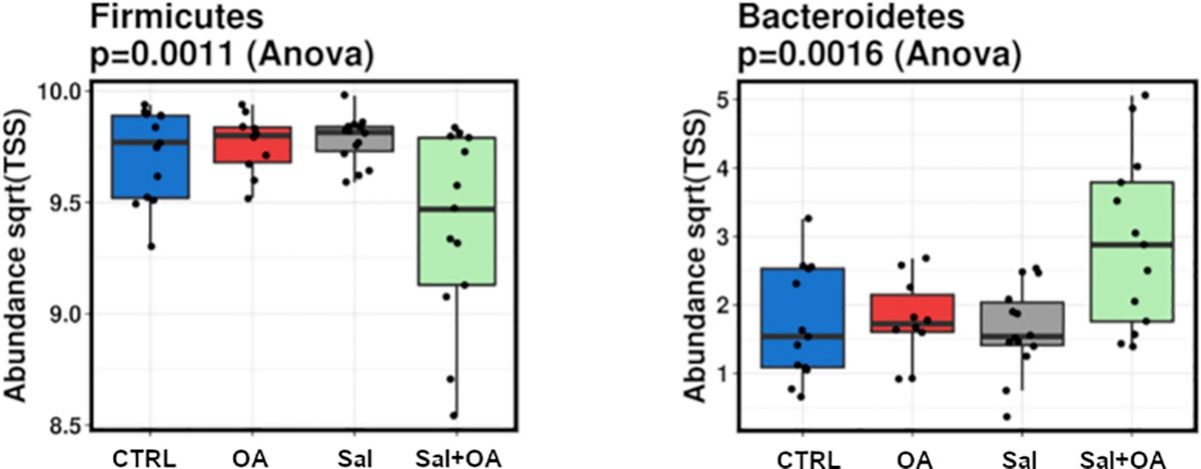
Significant univariate changes observed on phylum level in *S*. Typhimurium (Sal) challenge and organic acid (OA) treatments, CTR: Negative control.

**Fig 4:**
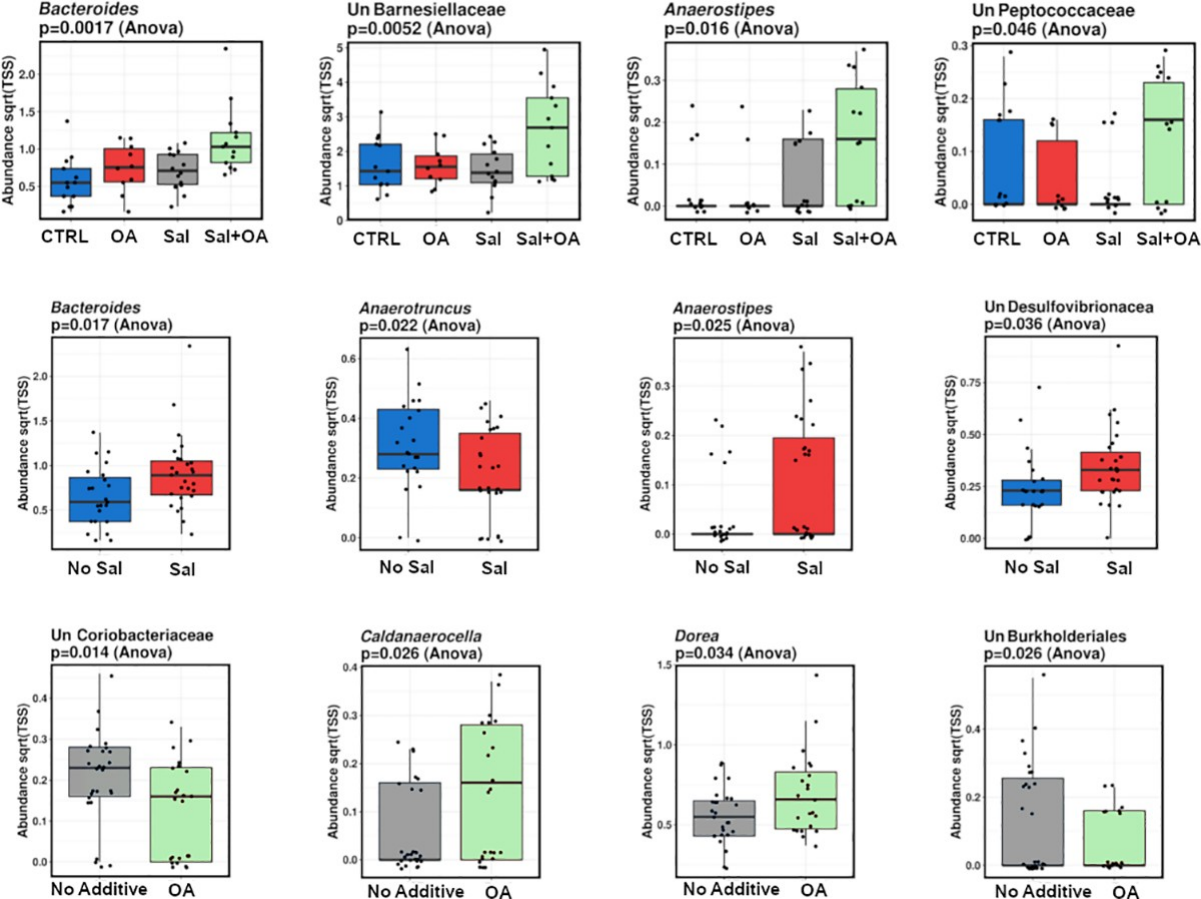
Genera abundances across treatment groups showing significant changes due to *S*. Typhimurium (Sal) challenge and/or organic acid (OA) administration.

We then examined the individual impact of taxa at various taxonomic levels. At a phylum level, we found a change of Bacteroidetes (*P* = 0.0016) and Firmicutes (*P* = 0.0011), (Fig 3). *S*. Typhimurium was associated with a marginal increase in Bacteroidetes (*P* = 0.069). At the genus level, *Bacteroides* and unclassified Barnesiellaceae were both increased in supplemented/challenged birds (*P* = 0.0017, *P* = 0.0052) respectively. *Anaerotruncus* declined in *S*. Typhimurium infected birds (*P<* 0.05) while *Bacteroides*, *Anaerostipes*, and unclassified Desulfovibrionaceae were significantly higher. OA supplementation was associated with a decline in unclassified Coriobacteriaceae and unclassified Burkholderiales, and an increase in *Caldanaerocella* and *Dorrea*, (P< 0.05) (Fig 4).

Although beta diversity analysis showed no significant alterations at an OTU level, there were numerous OTUs changed by different treatments, individually or in combination. A number of OTUs assigned to *Faecalibacterium prausnitzii* were highly significantly changed, however, cumulatively the genus *Faecalibacterium*, in this dataset comprised only from *F*. *prausnitzii* OTUs, was not significantly altered, indicating that this could be due to the split-OTU or strain rearrangement.

### Short-chain fatty acid production

**Fig 5:**
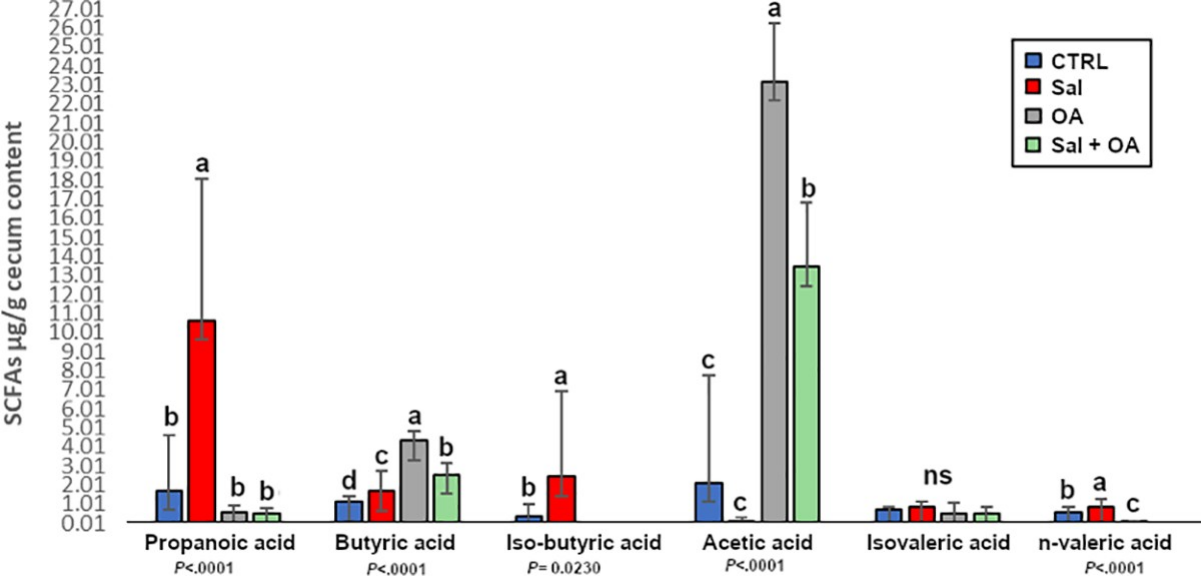
Impact of *S*. Typhimurium (Sal) challenge and OA administration on six SCFA concentrations in broiler chicken cecum. ^abcd^Means in the bar with different superscripts differ significantly. ns: not significant.

A significant rise in cecal propionic acid was observed in association with *S*. Typhimurium challenge against any of the other treatments. OA supplemented groups were characterized by a significant increase in acetic and butyric acid concentrations, along with a decrease in the concentration of propionic and n-valeric acids (Fig 5).

## Discussion

*S*. Typhimurium is a well-known enteric pathogen that can colonize bird’s gastrointestinal tract causing salmonellosis in broiler chickens [28,29]. Salmonellosis is strongly associated with negative influence on chicken growth performance parameters which could be regarded as an indication of the severity of the infection. Reports indicate that average body weight gain can decrease by 14% to 33%, and FCR to rise by 5% to 20% [30,31]. Our results show that organic acid supplementation marginally influenced the overall performance following *Salmonella* challenge. This could be influenced by the sample size, and it is quite possible that the marginal improvement would become significant if we had a higher power. Abudabos and Al-Mufarrej [32] reported that the addition of essential oils and other phytogens with OA, were as efficient as an antibiotic regarding growth performance and feed utilization; therefore it is possible that other similar products can amplify the marginal improvements we detected.

Chicken GIT has two primary functions which frequently interfere with each other: nutrient absorption and utilization and defence against enteric pathogens. Normal microbiota plays a vital role in supporting host physiological homeostasis [33] and contributes to chicken’s susceptibility to bacterial infection [11]. Although several individual taxa were significantly changed in our dataset, multivariate analysis shows that total microbial community was not significantly altered by *S*. Typhimurium induced infection, which is consistent with previous reports in *S*. Enteritidis challenged chicken [34–36]. However, we found that organic OA significantly alter microbial community of birds at a phylum (*P* = 0.008) and genus level (*P* = 0.03). Comparatively, recent reports show that the broiler diet supplementation with organic acids-based formulation influences intestinal microbiota and activates the expression of gut barrier genes [37].

It was hypothesized that the use of organic acids in poultry could reduce the abundance of lactic acid bacteria present in the bird’s intestine, thereby increasing the chance of *Salmonella sp*. colonizing. This particularly may occur as organic acids can be restricted to the crop and may not be capable of handling a high inclusion of *Salmonella* [7] in the gut. Nevertheless, our performance data did not indicate any reduction in broilers ability to utilize feed and nutrients in terms of bird’s performance over the entire span of the trial. SCFA-producing genus, *Bacteroides* was affected by the treatments [38].

The high abundance of unclassified Barnesiellaceae was observed in supplemented and challenged birds (T4), a previous study has reported that increased Barnesiellaceae in large intestinal contents was linked to tibial dyschondroplasia (TD), which is considered one of the most serious nutritional and metabolic disorders in broilers [39]. However, this increase did not occur in individual treatment, OA (T2) or *Salmonella* (T3), indicating their combination may promote unclassified Barnesiellaceae. *Anaerostipes* were substantially increased in all challenged birds; similar results have been reported previously in *S*. Enteritidis inoculation birds [36]. The *Anaerotruncus* genus declined in *S*. Typhimurium infected birds; however, Liu et al. [36] reported opposing results in *S*. Enteritidis challenged chicken. Organic acid blend supplementation was linked to a decline in unclassified Coriobacteriaceae and unclassified Burkholderiales; similar effects were reported in tetracycline and streptomycin treated laying hens [40].

Dittoe et al. [41] showed that organic acids supplementation in poultry demonstrated competence to improve bird’s performance by changing the pH of the GIT consequently alter microbiota composition by eliminating pH-sensitive pathogens and enhancing the morphology of intestine [41]. Villi length is associated with good intestinal health and high absorptive capacity [42]. Several studies proposed that organic acids administration increases villus length in poultry [43,44]. Results from the present study show that *S*. Typhimurium infection was linked to decreased intestinal surface area and villus length, villus fusion, and flattening with a high degree of villus deterioration.

Organic acid blend supplementation resulted in improved villi histomorphometric measurements where the birds challenged and supplemented with organic acid blend had significantly higher villus length than the challenged group. It was previously reported that a significant increase in *Faecalibacterium prausnitzii* correlated with intestinal epithelial health due to its strong metabolite production, particularly butyrate [45], which also applies for human health. In our data genus *Faecalibacterium* was one of the most abundant genera, and significant, yet inconclusive, strain level alterations we noted cannot be reliably associated with the histological changes. Fysal® Fit 4 has been reported to improve the morphology of the ileum and lower cumulative FCR [31].

SCFAs play a powerful role in host-pathogen interactions by multiple regulatory functions for instant regulation of virulence gene in some enteric pathogens through chemical environment modification as a form of acid stress, in addition to a broad impact on host energy homeostasis [46]. SCFAs are secreted in the ceca by bacterial fermentation of complex non-digestible polysaccharides. Acetic, butyric and propionic acids are the most abundant SCFAs accounting for more than 90% of colonic SCFAs [47]. Our results show that the highest concentration of butyric, *i-*butyric and acetic acids were observed in organic acid supplemented broilers. Acetic acid and butyric acid have been reported to positively influence the host's energy state by providing a carbon source for intestinal microbiota through the activation of glyoxylate pathway enzymes [48–50].

Some concerns for the use of organic acids as AGPs alternatives include their possible ability to influence pH and the inability to effectively reach the lower GIT, and their predicted hindering influence on beneficial bacteria such as lactic acid bacteria. For example, Thompson and Hinton [7] noted that as SCFAs pass through the digestive tract, their concentration declines because of digestion and metabolism. Our data show that organic acid supplementation improved cecal SCFAs, which could be related to the promotion of SCFA producing microbiota, without altering major probiotic taxa such as *Lactobacillus*.
